# TOWARD STRUCTURAL CHANGE: ADVANCING PRAXIS ON AGE-FRIENDLY CITIES AND COMMUNITIES

**DOI:** 10.1093/geroni/igac059.554

**Published:** 2022-12-20

**Authors:** Emily Greenfield

**Affiliations:** Rutgers, The State University of New Jersey, New Brunswick, New Jersey, United States

## Abstract

The global age-friendly cities and communities (AFCC) movement has inspired leaders across the public, private, and academic sectors to re-imagine how environments can better support long and healthy lives. There is growing recognition, however, of structural and other constraints that impede the translation of AFCC aspirations into systematic, comprehensive, impactful, and sustainable action. This paper will describe the importance of researchers’ engagement in AFCC praxis to better support the movement toward structural change for aging equity and healthy aging. The paper will present a case example of academic researchers’ long-standing involvement in collaborative efforts toward the development and sustainability of age-friendly community initiatives in New Jersey. The case will emphasize the importance of creating synergies across research, teaching, and service activities; cultivating coordinated AFCC efforts across the mico, meso, and macro levels; and developing sustainable structures for deliberate inter-organizational and multi-sectoral partnerships toward short- and long-term goals.

